# Identification of a Modified *HOXB9* mRNA in Breast Cancer

**DOI:** 10.1155/2020/6065736

**Published:** 2020-02-13

**Authors:** Ayako Nakashoji, Tetsu Hayashida, Yuko Kawai, Masayuki Kikuchi, Rurina Watanuki, Takamichi Yokoe, Tomoko Seki, Maiko Takahashi, Kazuhiro Miyao, Shigeo Yamaguchi, Yuko Kitagawa

**Affiliations:** Department of Surgery, Keio University School of Medicine, Shinanomachi 35 Shinjuku-ku, Tokyo 160-0016, Japan

## Abstract

First identified as a developmental gene, *HOXB9* is also known to be involved in tumor biological processes, and its aberrant expression correlates with poor prognosis of various cancers. In this study, we isolated a homeodomain-less, novel *HOXB9* variant (*HOXB9v*) from human breast cancer cell line-derived mRNA. We confirmed that the novel variant was produced from variationless *HOXB9* genomic DNA. RT-PCR of mRNA isolated from clinical samples and reanalysis of publicly available RNA-seq data proved that the new transcript is frequently expressed in human breast cancer. Exogenous HOXB9v expression significantly enhanced the proliferation of breast cancer cells, and gene ontology analysis indicated that apoptotic signaling was suppressed in these cells. Considering that HOXB9v lacks key domains of homeobox proteins, its behavior could be completely different from that of the previously described variationless HOXB9. Because none of the previous studies on HOXB9 have considered the presence of HOXB9v, further research analyzing the two transcripts individually is warranted to re-evaluate the true role of HOXB9 in cancer.

## 1. Introduction

Homeobox (HOX) genes were initially characterized as developmental genes, which code for transcription factors that play critical roles in embryogenesis. Evolutionarily, they are highly conserved and share a high degree of homology, especially within the same paralog groups. All 39 mammalian HOX genes consist of two exons and a single intron. The homeobox domain, encoded in the second exon [1], includes the DNA binding site. The diverse and specific transcriptional activities of the HOX proteins often depend on key cofactors including PBX, MEIS, and PREP, which interact with hexapeptide motifs of HOX proteins [2].

HOX genes play key roles in both solid and hematological malignancies, including cancers of the colon, breast, prostate, lung, brain, thyroid, ovary, bladder, kidney, skin, and blood [3, 4]. HOXB9, the ninth paralog in the HOX-B cluster, is associated with the growth and progression of multiple cancers. In lung adenocarcinoma, HOXB9 promotes metastasis by activating the WNT signaling pathway [5, 6]. In breast cancer, the gene induces the expression of proangiogenic factors, increasing the cell motility and supporting epithelial-mesenchymal transition (EMT) [7, 8]. HOXB9 also promotes the growth of colon cancer by activating IL6 signaling, inducing the secretion of angiogenic factors and increasing proliferation of tumor cells [9]. Similar observations are found in ovarian cancer and hepatocellular carcinoma [10, 11]. Thus, HOXB9 activates the WNT signaling pathway and enhances the acquisition of capabilities critical to the transformation of normal cells to cancer, including EMT and the growth of new vasculature within the tumor microenvironment.

HOXB9 is also directly associated with cancer-induced patient mortality. The duration of disease-free and overall survival of patients with HOXB9-positive breast cancer is significantly shorter compared with patients with HOXB9-negative breast cancer [12]. Increased HOXB9 expression significantly correlates with decreased overall survival for patients with colorectal cancer [9]. Patients of laryngeal squamous cell carcinoma, hepatocellular carcinoma, glioma, and endometrial cancer also present poor outcomes or tumor progression, resulting from aberrant HOXB9 expression [13–16].

The recent elucidation of the critical and diverse roles HOXB9 plays in various cancers have led us to explore the mechanism of this gene's role in cancer progression. In the present study, we identified and characterized a *HOXB9* variant (*HOXB9v*) of mRNA from human breast cancer cell lines. The sequence of *HOXB9v* largely differs from the previously known *HOXB9* normal transcript (*HOXB9n*), and we found it lacks some important domains of HOX genes. Based on these findings, we inferred its role and function were different from H*OXB9n*. This study aimed to confirm the presence of HOXB9v in clinical breast cancer specimens and investigate the role of HOXB9v in breast cancer progression.

## 2. Materials and Methods

### 2.1. Cell Culture

We cultured eight human breast cancer cell lines (MCF7, MDA-MB-231, MDA-MB-468, Hs578T, HCC38, BT-474, BT-549, and SKBR3), a human colon cancer cell line (WiDR), and a mouse breast cancer cell line (4T1) in DMEM supplemented with 10% FBS (Thermo Fisher Scientific, Waltham, MA, USA) with the addition of antibiotic and antimycotic agent (antibiotic-antimycotic mixed stock solution, Nacalai Tesque, Inc., Kyoto, Japan). T47D cells were grown in RPMI1640 medium with 10% FBS and antibiotic and antimycotic agents. MCF10A cells were grown in DMEM/F12 (1 : 1) medium with 5% horse serum (Thermo Fischer Scientific), 20 ng/mL EGF (PeproTech, Rocky Hill, NJ, USA), 0.5 mg/mL hydrocortisone, 100 ng/mL cholera toxin, 10 *µ*g/mL insulin (all Sigma-Aldrich, St. Louis, MO, USA), and antibiotic and antimycotic agents. Cells were maintained at 37°C in a humidified 5% CO_2_ incubator.

### 2.2. Patients and Samples

Clinical specimens of human breast cancer (*n* = 14) were collected from patients with primary operable breast cancer who underwent total or partial mastectomy between July and November 2018 in Keio University Hospital (Tokyo, Japan). Patient-matched healthy breast epithelium samples (*n* = 6) were collected from healthy breast tissue from patients who underwent total mastectomy. Ethics approval for the present study was provided by the Ethics Committee at the Keio University School of Medicine (approval number: 20180090), and the study was performed in accordance with the provisions of the Declaration of Helsinki (as revised in Fortaleza, Brazil, October 2013). All included patients gave informed consent.

### 2.3. mRNA and Genomic DNA Extraction from Cell Lines and Clinical Specimens

Total RNA and genomic DNA were extracted using the RNeasy Mini Kit and QIAmp DNA Mini Kit (Qiagen, Hilden, Germany) following the manufacturer's instructions. Clinical specimens were homogenized using a Minilys homogenizer (Bertin Instruments, Bretonneux, France) using 2.4 mm metal beads prior to mRNA extraction. Total RNA was converted to cDNA using the High-Capacity RNA-to-cDNA Kit (Thermo Fischer Scientific).

### 2.4. HOXB9 Cloning

HOXB9 transcripts and genomic DNA were amplified by PCR and subsequently cloned into the pME-HA vector (Lucigen, Middleton, WI, USA) using the Expresso CMV Cloning & Expression system (Lucigen). The primers used were as follows: sense 5′ GAAGGAGATACCACCATGTCCATTTCTGGGACGCTTAGC 3′ and antisense 5′ GGGCACGTCATACGGATACTCTTTGCCCTGCTCCTTATT 3′.

After transformation into Competent Quick DH5*α* cells (Toyobo, Osaka, Japan) and culturing in kanamycin-containing LB plates, at least 4 colonies were selected for each sample.

### 2.5. Sequence Analysis

Sequences were analyzed using the BigDye Terminator V3.1 Cycle Sequencing kit (Thermo Fisher Scientific) and Applied Biosystems 3500 Genetic analyzer (Thermo Fisher Scientific). The GENETYX-MAC Ver.19 software (GENETYX, Osaka, Japan) was used for homology alignment.

### 2.6. RT-PCR Analysis

The expressions of *HOXB9n* and *HOXB9v* were detected by RT-PCR using the following primers: sense, 5′ TGTCCATTTCTGGGACGCTT 3′; antisense, 5′ CTACGGTCCCTGGTGAGGTA 3′. The genomic DNA of *HOXB9* was detected by PCR using the following primers: sense, 5′ CGAGAGAGCTGCAAGTCGAT 3′; antisense, 5′ CTGCCGTCCGTCTACCAC 3′. The primers for genomic DNA were designed against exon 1 and the intron region of the *HOXB9* gene to ensure that the pair will specifically amplify only genomic DNA and not cDNA derived from mRNA. The conditions applied for amplification were as follows: 94°C for 1 minute, followed by 35 cycles at 95°C for 5 seconds, 55°C for 5 seconds, and 72°C for 5 seconds, and run on the Life ECO thermal cycler (Hangzhou Bioer Technology, Hangzhou, China) using the SapphireAmp Fast PCR Master Mix (Takara Bio, Shiga, Japan) or KOD-Plus-Neo (Toyobo).

### 2.7. Public Data Reanalysis

The RNA sequence data set (GSE119937) in FASTQ format was downloaded via SRA (SRP161704) using the SRA Toolkit (version 2.3.4-2). RNA-seq reads were aligned by STAR (version 2.6.1b) against the hg38 reference genome. All reads mapped on the *HOXB9* gene were visually confirmed by taking snapshots in IGV (version 2.4.15).

### 2.8. Establishment of Stable MCF7 Cell Lines Overexpressing HOXB9n or HOXB9v

The *HOXB9n* and *HOXB9v* sequences were amplified by PCR and subsequently cloned into the pBiT3.1-N [CMV/HiBiT/Blast] expression vector (Promega, Tokyo, Japan) at the Xhol and BamHI sites using Ligation high Ver.2 (Toyobo). The primers used for amplification were as follows: sense, 3′ ATACCTCGAGGTCCATTTCTGG 5′; antisense, 3′ CACGTCATACGGATCCTCTTTG 5′. MCF7 cells were transfected with the HiBiT-tagged *HOXB9n* or *HOXB9v* vector using the Viafect Transfection reagent (Promega) and selected with 10 *µ*g/mL of blasticidin for at least 4 weeks. The expression of HiBiT-tagged HOXB9n and HOXB9v proteins was detected using the Nano-Glo HiBiT Blotting System (Promega) as per the manufacturer's instructions.

### 2.9. Transient Overexpression of HOXB9n and HOXB9v in MDA-MB-468 Cells

MDA-MB-468 cells were transfected with aforementioned HiBiT-tagged *HOXB9n* or *HOXB9v* vector using the jetPRIME (Polyplus-transfection, Illkirch, France) as per the manufacturer's instructions.

### 2.10. Quantitative Real-Time PCR

Quantitative real-time PCR was run on ViiA7 (Thermo Fisher Scientific) using Fast SYBR Green Master Mix (Thermo Fisher Scientific). Preincubation was performed for 20 seconds at 95°C and amplification for 41 cycles (1 second of denaturation at 95°C and 20 seconds of annealing and extension at 60°C), followed by melt-curve analysis. *GAPDH* served as an internal control, and QuantStudio Real-Time PCR Software v1.2 (Thermo Fisher Scientific) was used for quantification. The relative standard curve method was used for linear regression analysis of unknown samples, and data are presented as fold change between samples. The primers used were as follows: *HOXB9*: sense, 5′ CGGTGGCTGTCGTGAAATT 3′; antisense, 5′ CGAGACAATCACCCCCAAAG 3′; *GAPDH*: sense, 5′ATCATCCCTGCCTCTACTGG 3′; antisense, 5′ TTTCTAGACGGCAGGTCAGGT 3′.

### 2.11. Cell Proliferation Assay

Cell proliferation in three-dimensional (3D) culture was measured using the 24-well Bio-Assembler kit and NanoShuttle-PL (Greiner Bio-One, Kremsmünster, Austria). Further, 20,000 cells/well were incubated for 48 hours before taking photomicrographs.

Cell proliferation in flat culture was measured using Cell Count Reagent SF (Nacalai Tesque). Briefly, 5000 cells/well in 96-well microtiter plates (Sumilon, Sumitomo Bakelite, Tokyo Japan) were incubated for 5 days or after 24 hour continuous exposure to either 30 nM HXR9 or 30 nM CXR9 for 4 days. The absorbance in the wells was measured on days 1, 3, and 5 using a Sunrise Rainbow-RC (TECAN, Männedorf, Switzerland) microplate spectrophotometer at 450 nm, using 600 nm as reference. The HXR9 (WYPWMKKHHRRRRRRRRR-) and control CXR9 (WYPAKKHHRRRRRRRRR) peptides were synthesized by Eurofins Genomics K. K. (Tokyo, Japan).

### 2.12. Microarray and Differential Expression Analyses

Total RNA was isolated using the RNeasy Mini kit (Qiagen). Microarray was performed using the human Clariom S assay (Thermo Fisher Scientific) by the GeneChip Scanner 3000 7G system (Affymetrix, Santa Clara, CA, USA), and the results were analyzed by TAC 4.0 software (Thermo Fisher Scientific). Genes having a false discovery rate (FDR) under 0.05 and upregulated in HOXB9v samples were considered as upregulated differentially expressed genes (up-DEGs). DAVID Bioinformatics Resources 6.8 [17] was used for gene ontology and pathway analysis of DEGs. R ver. 3.5.0 software was used to draw the heatmap of 37 genes included in GO:0043069 (negative regulation of programmed cell death).

## 3. Results

### 3.1. Identification of a Novel Transcript HOXB9v, Which Lacks Important Domains of HOX Gene

**Figure 1:**
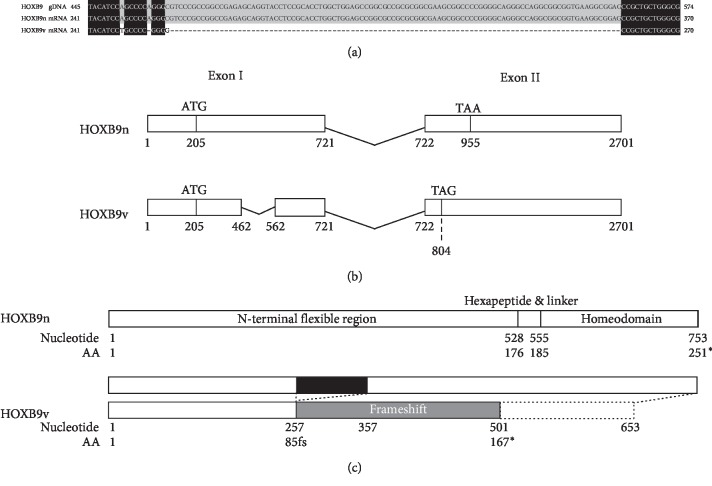
Structure and sequence of *HOXB9n* and *HOXB9v*. (a) Sequences of genomic DNA *HOXB9*, mRNA *HOXB9n*, and mRNA *HOXB9v* (black and grey highlighting indicates homology between sequences). (b) Schematic diagram of *HOXB9n* (upper) and *HOXB9v* (lower) transcripts showing the exons, splicing regions, and the deleted region. In *HOXB9v*, a 100 bp deletion in exon 1 leads to a frameshift and a stop codon formation (TAA). (c) Protein structure of HOXB9n and HOXB9v. Transcription from the start codon (ATG) to the stop codon (TAA) results in the translation of a full-length HOXB9n protein (upper). The 100 bp deletion in HOXB9v (shown in black) leads to a frameshift from AA85 (shown in grey) and truncation of protein coding by a stop codon (TAG) at AA167, which results in HOXB9v protein without hexapeptide and homeodomain.^*∗*^Stop codon.

We isolated total RNA from human breast cancer cell lines (MCF7, T47D, MDA-MB-231, MDA-MB-468, HCC38, BT-474, BT-549, Hs578T, and SKBR3), a normal human mammary gland cell line (MCF10A), and a human colon cancer cell line (WiDR). We cloned and sequenced the *HOXB9* gene from the nucleic acids derived from these cell lines. We isolated a novel variant of the *HOXB9* transcript from the MCF7, T47D, MDA-MB-231, MDA-MB-468, Hs578T, HCC38, and MCF10A cell lines. The sequence homology of the new transcript is shown in Figures 1(a) and [Supplementary-material supplementary-material-1].

We will refer to the new truncated transcript as *HOXB9v*, to distinguish from the full-length *HOXB9* transcript (*HOXB9n*). A 100-base deletion in exon 1 in the new transcript leads to a frameshift and the formation of a stop codon (TAG), which truncates the protein coding at AA167.  Figure 1(b) is a schematic diagram of *HOXB9* transcripts showing the exons and the deleted lesion. The encoded protein will therefore possibly lack the homeobox domain, DNA binding domain, and the hexapeptide motif, a major player in cofactor interactions (Figure 1(c)). The *HOXB9v* sequence has been submitted to GenBank under Accession No. LC466645.

**Figure 2:**
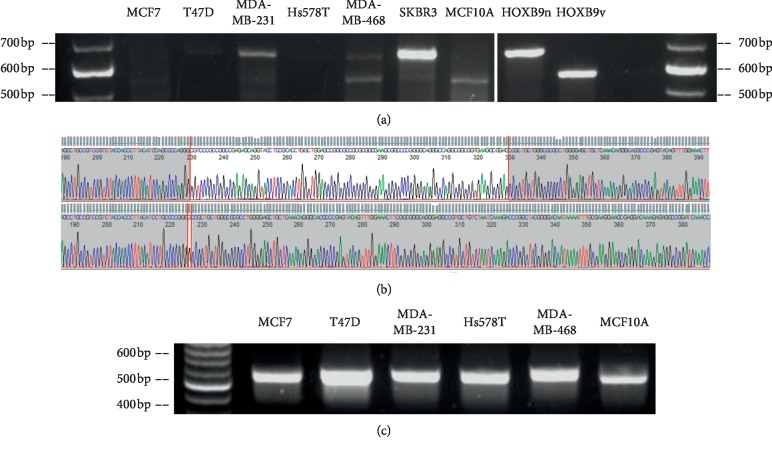
Detection of mRNA and genomic DNA of *HOXB9* in cell lines. (a) Both *HOXB9n* and *HOXB9v* transcripts were detected in breast cancer cell line mRNA (cDNA). (b) Sequencing confirmation of PCR product of Figure 2(a); SKBR3 (*HOXB9n*, upper column) and MCF10A (*HOXB9v*, lower column). (c) No genomic variation was detected in genomic DNA of breast cancer cell line genomic DNA. *HOXB9v* transcripts are commonly found in human breast cancer specimens.

We next performed PCR analyses to verify the presence of the *HOXB9v* transcript and identify genomic DNA variations of the *HOXB9* gene in human breast cancer cell lines. The primer target region included the deletion site of *HOXB9v* and the amplicon for *HOXB9n* was 643 bp and 543 bp for *HOXB9v*. We detected both *HOXB9n* and *HOXB9v* transcripts in breast cancer cell lines (Figure 2(a)). The PCR products were sequenced and were confirmed that each band corresponded to the exact sequence of the *HOXB9n* or *HOXB9v* (Figure 2(b)). By PCR of breast cancer cell lines' genomic DNA, no bands indicative of genomic DNA variations in *HOXB9* were detected. The amplicon of variation-less *HOXB9* genomic DNA was 569 bp (Figure 2(c)). Sequencing of the PCR products confirmed that they had no variations or deletions in genomic DNA ([Supplementary-material supplementary-material-1]). These findings show that the two transcripts (*HOXB9n* and *HOXB9v*) were produced from variation-less *HOXB9* genomic DNA.

**Figure 3:**
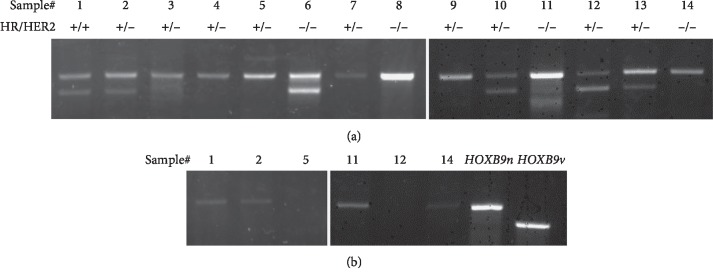
Detection of *HOXB9v* in clinical samples. (a) *HOXB9n* and *HOXB9v* were detected in breast cancer clinical samples. (b) *HOXB9v* was not detected in normal mammary gland samples. Hormone receptor status and HER2 status of each cancer sample are shown beneath each sample number. We assigned the same sample number if cancer and normal gland samples were acquired from the same patient.

To determine the presence of *HOXB9v* transcripts in human breast cancer samples, we performed PCR analysis. *HOXB9v* was commonly detected from clinical breast cancer samples (Figure 3(a)), regardless of their hormone receptor and HER2 status. However, *HOXB9v* was not detected in normal mammary gland samples (Figure 3(b)).

**Figure 4:**
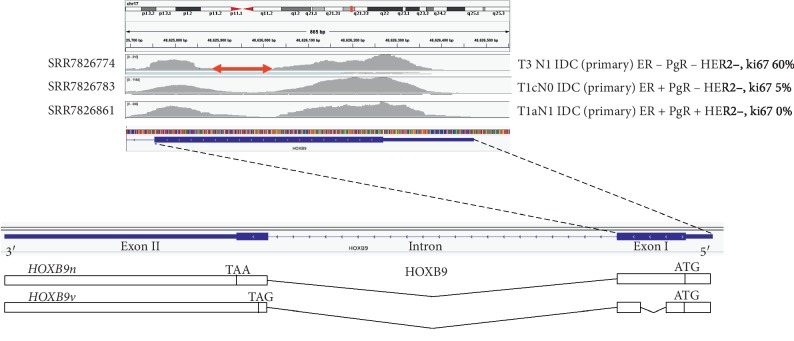
NGS data indicates the presence of *HOXB9v* in breast cancer. Showing RNA sequence breast cancer sample data (SRR782677) mapped on *HOXB9* exon 1. The blue band on the bottom shows the coding region (the thick part) and the 5′ UTR (the thin part) of exon 1. There is a region where data are sparsely mapped (indicated with the red arrow), which matches the deletion region in *HOXBv*. HOXB9v overexpressing MCF7 and MDA-MB-468 cells proliferated faster compared with HOXB9n overexpressing cells.

Thus, to further confirm the presence of *HOXB9v* in human breast cancer samples, we reanalyzed a publicly available breast cancer RNA sequence data set (GSE119937) [18] and mapped the reads onto the *HOXB9* gene sequence. We identified a region in exon 1 where the number of mapped reads was low in numerous samples; this region matched the deletion region of *HOXB9v* (Figure 4).

**Figure 5:**
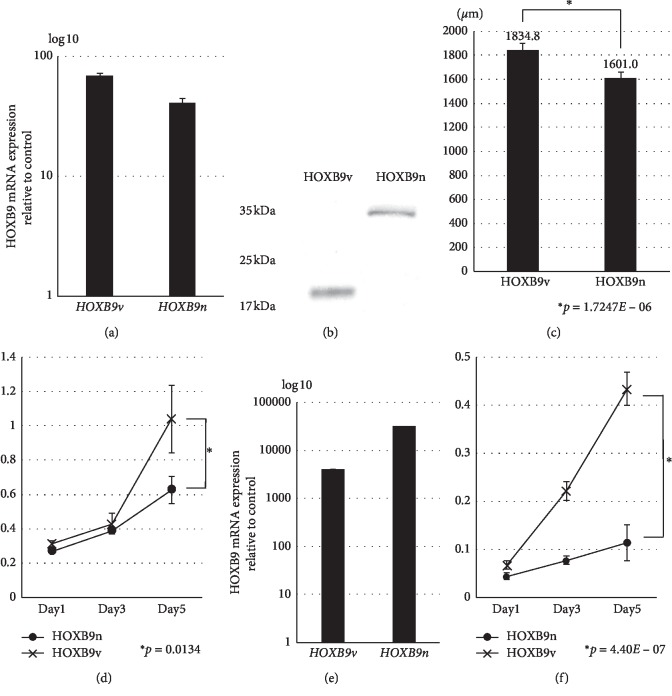
(a–d) Confirmation and proliferation of stable MCF7 cell lines that overexpress HOXB9n or HOXB9v. Levels of HOXB9n and HOXB9v (a) mRNA and (b) protein in stable cell lines. Proliferation of the HOXB9n and HOXB9v stable cell lines using a (c) 3D cell culture model and a (d) 2D cell culture model. (e-f) Confirmation and proliferation of MDA-MB-468 cells with transient overexpression of *HOXB9n* or *HOXB9v*. (e) Levels of *HOXB9n* and *HOXB9v* mRNA expression. (f) Proliferation of *HOXB9n* and *HOXB9v* overexpressing cells by 2D cell culture model.

To determine the role of HOXB9v in breast cancer, we established stable MCF7 cell lines overexpressing HOXB9n or HOXB9v. Gene and protein expressions of HOXB9n and HOXB9v were verified in both cell lines (Figures 5(a) and 5(b)). Cell proliferation assays in both 3D culture and flat culture showed that HOXB9v overexpression increased MCF7 cell growth (Figures 5(c) and 5(d)). We also transiently overexpressed *HOXB9n* or *HOXB9v* in MDA-MB-468 cells, and the cell proliferation assay showed that *HOXB9v* overexpression increased MDA-MB468 cell growth (Figures 5(e) and 5(f)).

### 3.2. HXR9 and CXR9 Treatment

**Figure 6:**
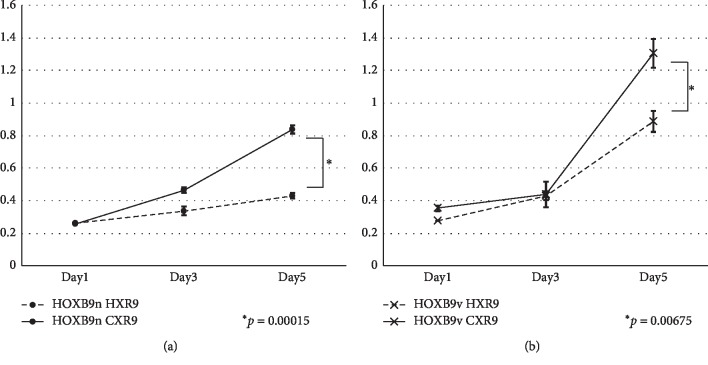
(a) HOXB9n-MCF7 cells and (b) HOXB9v-MCF7 cells were treated with HXR9 or with a control peptide, CXR9. HXR9 significantly inhibited the proliferation of both cell lines.

HXR9, an 18-amino acid peptide, competently inhibits the hexapeptide motif of HOX proteins and prevents HOX-PBX binding [19]. HOXB9n and HOXB9v expressing MCF7 cells were treated with HXR9 or a control peptide, CXR9 (Figures 6(a) and 6(b)). Although HOXB9v lacks the hexapeptide motif, which is known to interact with PBX proteins [20], HXR9 significantly inhibited proliferation of both cell lines.

### 3.3. Microarray and Gene Ontology Analysis

**Figure 7:**
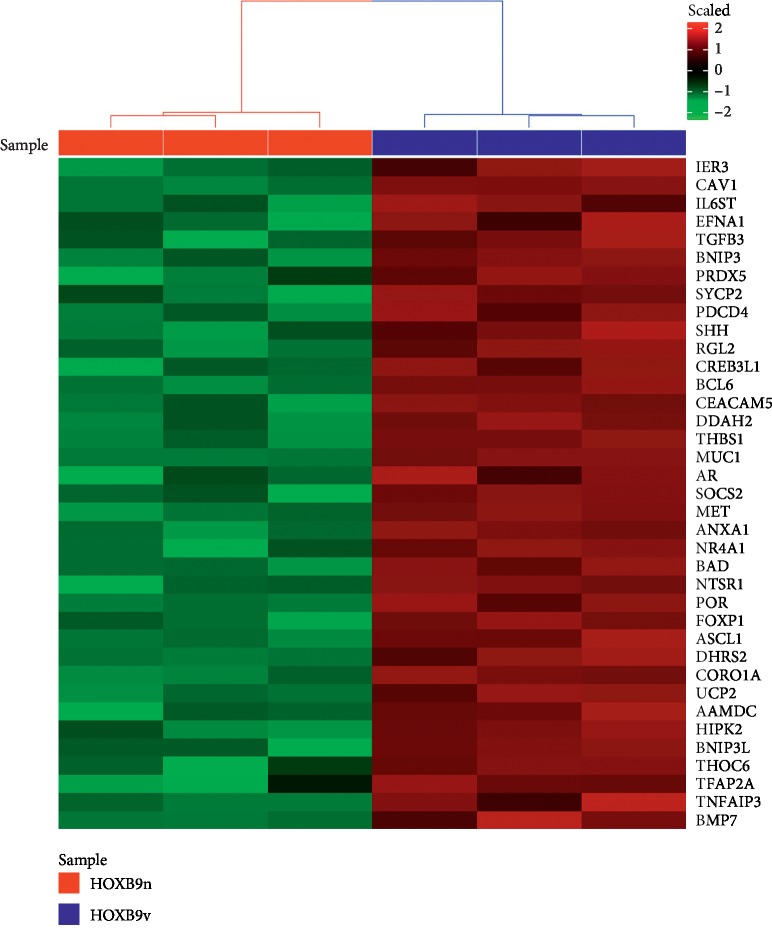
Microarray data heatmap of HOXB9n-MCF7 and HOXB9v-MCF7. Genomic heatmap compares expression of 37 genes which are involved in suppression of apoptotic process (GO:0043069), with red and green color intensities indicating high and low expressions, respectively. Programmed cell death is suppressed in HOXB9v-MCF7 cells.

To explore the reason behind the faster proliferation of HOXB9v overexpressing cells, we performed microarray and gene ontology analyses using HOXB9n and HOXB9v-expressing MCF7 cells. We chose 1056 genes as DEGs, and gene ontology analysis showed that up-DEGs between HOXB9n and HOXB9v expressing cells presented significant differences in pathways relevant to apoptosis suppression (GO:0060548∼negative regulation of cell death, GO:0043069∼negative regulation of programmed cell death, and GO:0043066∼negative regulation of apoptotic process) and steroid hormone response (GO:0048545∼response to steroid hormone, GO:0032870∼cellular response to hormone stimulus, and GO:0071383∼cellular response to steroid hormone stimulus) (Table 1). The genomic expression heatmap comparing 37 genes involved in the apoptotic process (GO:0043069∼negative regulation of programmed cell death) shows that apoptosis is highly suppressed in HOXB9v expressing cells (Figure 7).

## 4. Discussion

In the present study, we identified a novel modified transcript of *HOXB9*, in which a deletion in exon 1 causes a frameshift, formation of a stop codon, and truncation of protein coding. This leads to a defect in the homeodomain and hexapeptide regions, which are both crucial for HOX gene function. We confirmed that *HOXB9v* is widely present in human breast cancer cell lines and clinical breast cancer samples, however not in normal gland samples. Detection of *HOXB9v* in MCF10A may be attributed to the fact that this cell line is not karyotypically normal, while it maintains major characteristics of normal breast epithelium [21]. We further confirmed that *HOXBv* and the previously known *HOXB9n* were both produced from variationless *HOXB9* genomic DNA. Two distinct mRNA products from a single variationless genomic DNA may indicate that *HOXB9v* is a novel splice variant of *HOXB9*. It may be assumed that *HOXB9v* has a typical splice site of GU as a 5′ donor site and AG as a 3′ acceptor site [22]; however, the single-nucleotide transitions at 248 and 258 and the single-nucleotide deletion at 254 must be taken into account. Further investigation and discussions are needed to decide whether *HOXB9v* is a splice variant or an mRNA modified by a different mechanism. Nonetheless, the modified transcript is certainly not a result of variations in genomic DNA. Additionally, *HOXB9v* results in a different stop codon site from that of *HOXB9n* and may become a target of nonsense-mediated mRNA decay (NMD), an mRNA surveillance mechanism that eliminates premature translation-termination codons. However, NMD is known to be initiated if an exon-junction complex is present more than 50–55 bases downstream of the stop codon [23]. *HOXB9v* forms its exon-junction complex upstream from its stop codon; therefore, we infer it escapes from NMD.

Nevertheless, the production of splice variants of HOX genes lacking the homeodomain is likely a common phenomenon. Murine Hoxb9, which shares sequence similarity with human HOXB9, is reported to generate a splice variant without a homeodomain. Other HOX genes, including human *HOXA1, HOXB6, HOXA9, HOXA10*, murine *Hoxb6* and *Meis1*, and Xenopus *XlHbox2*, have also been reported to generate splice variants lacking homeodomains [24–28]. Interestingly, *HOXA9T*, a homeodomain-less isoform of *HOXA9*, which is structurally similar to *HOXB9v*, has been demonstrated to act as an oncogene in leukemia without directly binding to DNA [24]. HOXB9 has been reported to promote tumorigenesis in various types of cancers; however, no previous research on HOXB9 has shown a mRNA variant of HOXB9. Considering that the structure of the HOXB9v protein differs from that of the previously studied HOXB9n protein and lacks important domains such as homeodomain and hexapeptide motif, its function is likely to differ from that of HOXB9n. Further study is required to re-evaluate the role of HOXB9 in cancer. HOXB9n and HOXB9v should be assessed separately.

In the growth assay, MCF7 and MDA-MB-468 cells expressing HOXB9v at high levels presented more rapid proliferation than did those expressing HOXB9v. It is meaningful that similar results were observed in two cell lines: one with hardly any expression of HOXB9n and HOXB9v (MCF7) and the other with high expression of both (MDA-MB-468). Fostered proliferation by HOXB9v may be attributed to the suppressed apoptosis observed in our microarray studies. Gene ontology analysis of HOXB9v-expressing cells compared to HOXB9n-expressing cells indicated significant upregulation of pathways related to apoptosis suppression, further underscoring its role in this regard. Several HOX genes have been reported to regulate apoptosis in cancer [29, 30], and HOX-regulated apoptosis is a general mechanism used during development to maintain metameric patterns [31, 32]. However, the role of HOXB9 in apoptosis is yet to be investigated.

The binding selectivity of HOX proteins is influenced by cofactors, including members of the PBX, MEIS, and PREP families [2]. Additionally, HOX-PBX interactions involve a short HOX protein motif, the hexapeptide, located upstream of the homeodomain [33]. HXR9 is an 18-amino acid peptide and suppresses tumor proliferation by inhibiting HOX-PBX binding [19]. In our proliferation assay, HOXB9v expressing cell lines were sensitive to the HOX-PBX inhibitor HXR9, even though HOXB9v lacks the hexapeptide. This may be because HOXB9v, in consort with HOXB9n or other HOX proteins, indirectly promotes HOX-PBX binding.

## 5. Conclusions

We report a modified *HOXB9* mRNA variant that results in a homeodomain defect. We confirmed its presence in human breast cancer cell lines and breast cancer clinical samples and also revealed that *HOXB9v* may promote breast cancer proliferation by suppression of apoptosis. Further research is warranted to analyze HOXB9v and HOXB9n individually and re-evaluate the true role of HOXB9 in cancer.

## Figures and Tables

**Table 1:** Gene ontology analysis by differential gene expression. Apoptosis was suppressed in HOXB9v-MCF7 cells compared with HOXB9n-MCF7 cells.

Term	*P* value	Bonferroni
Negative regulation of cell death	7.36*E*^−07^	0.002961
Response to steroid hormone	2.24*E*^−06^	0.008994
Cellular response to zinc ion	2.66*E*^−06^	0.01065
Negative regulation of programmed cell death	3.30*E*^−06^	0.013228
Cellular response to hormone stimulus	6.93*E*^−06^	0.027551
Negative regulation of apoptotic process	7.26*E*^−06^	0.028853
Cellular response to steroid hormone stimulus	1.15*E*^−05^	0.045436
Negative regulation of transcription, DNA-template	1.17*E*^−05^	0.045904

## Data Availability

The HOXB9v sequence has been submitted to GenBank under Accession No. LC466645. The RNA sequence data set (GSE119937) used in public data reanalysis in the fastq format is available via SRA (SRP161704). Other datasets used and/or analyzed during the current study are available from the corresponding author on reasonable request.

## Supplementary Materials

Supplementary Materials Figure S1: full sequences of HOXB9n and HOXB9v mRNA (black highlight indicates the homology between sequences). Figure S2: sequence results of PCR products of breast cancer cell-line derived genomic DNA.
