# Supplementary material for: Identification of a Modified HOXB9 mRNA in Breast Cancer
Source: J Oncol. 2020 Feb 13;2020:6065736. doi: 10.1155/2020/6065736 (PMC7040399; doi:10.1155/2020/6065736)
Supplement: Supplementary Materials — Figure S1: full sequences of HOXB9n and HOXB9v mRNA (black highlight indicates the homology between sequences). Figure S2: sequence results of PCR products of breast cancer cell-line derived genomic DNA. [file 6065736.f1.docx]

Figure S1

Full sequences of *HOXB9n* and *HOXB9v* mRNA (black highlighting indicates homology between sequences).

HOXB9n 1 ATGTCCATTTCTGGGACGCTTAGCAGCTATTATGTCGACTCGATCATAAGTCACGAGAGTGAGGACGCGCCTCCAGCCAAGTTTCCTTCTGGCCAGTACGCGAGCTCGCGGCAGCCGGGC 120

HOXB9v 1 ........................................................................................................................ 120

HOXB9n 121 CACGCGGAGCACCTGGAGTTCCCCTCGTGCAGCTTCCAGCCCAAAGCGCCGGTGTTCGGCGCCTCCTGGGCGCCGCTGAGCCCGCACGCGTCCGGGAGCCTGCCGTCCGTCTACCACCCT 240

HOXB9v 121 ........................................................................................................................ 240

HOXB9n 241 TACATCCAGCCCCAGGGCGTCCCGCCGGCCGAGAGCAGGTACCTCCGCACCTGGCTGGAGCCGGCGCCGCGCGGCGAAGCGGCCCCGGGGCAGGGCCAGGCGGCGGTGAAGGCGGAGCCG 360

HOXB9v 241 .......T.....-...G---------------------------------------------------------------------------------------------------... 260

HOXB9n 361 CTGCTGGGCGCGCCTGGGGAGCTGCTCAAACAGGGCACGCCCGAGTACAGTTTGGAAACTTCGGCGGGCAGGGAGGCCGTGCTGTCTAATCAAAGACCCGGCTACGGGGACAATAAAATT 480

HOXB9v 261 ........................................................................................................................ 380

HOXB9n 481 TGCGAAGGAAGCGAGGACAAAGAGAGGCCGGATCAAACCAACCCCTCCGCCAACTGGCTGCACGCTCGCTCTTCCCGGAAAAAGCGCTGTCCCTACACCAAATACCAGACGCTGGAGCTA 600

HOXB9v 381 ........................................................................................................................ 500

HOXB9n 601 GAGAAGGAGTTTCTGTTCAATATGTACCTCACCAGGGACCGTAGGCACGAAGTGGCCAGACTCCTCAATCTGAGTGAGAGACAAGTCAAAATCTGGTTTCAGAACCGGCGGATGAAAATG 720

HOXB9v 501 ........................................................................................................................ 620

HOXB9n 721 AAGAAAATGAATAAGGAGCAGGGCAAAGAGTAA 753

HOXB9v 621 ................................. 653

Figure S2 Sequence results of PCR products of breast cancer cell-line derived genomic DNA

HOXB9 gDNA 445 TACATCCAGCCCCAGGGCGTCCCGCCGGCCGAGAGCAGGTACCTCCGCACCTGGCTGGAGCCGGCGCCGCGCGGCGAAGCGGCCCCGGGGCAGGGCCAGGCGGCGGTGAAGGCGGAGCCGCTGCTGGGCG 574

HOXB9n mRNA 241 TACATCCAGCCCCAGGGCGTCCCGCCGGCCGAGAGCAGGTACCTCCGCACCTGGCTGGAGCCGGCGCCGCGCGGCGAAGCGGCCCCGGGGCAGGGCCAGGCGGCGGTGAAGGCGGAGCCGCTGCTGGGCG 370

HOXB9v mRNA 241 TACATCCTGCCCC-GGGG---------------------------------------------------------------------------------------------------CCGCTGCTGGGCG 270


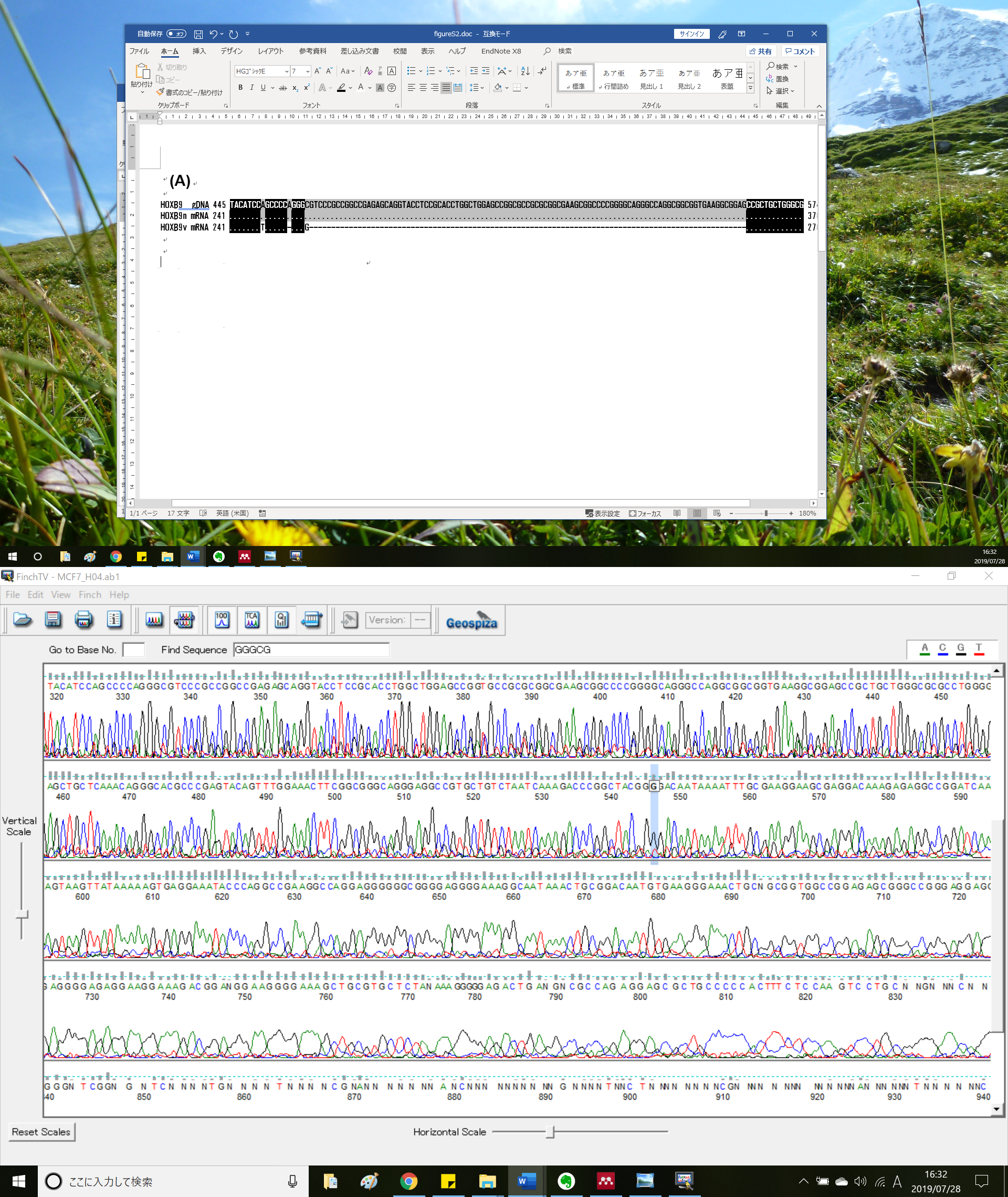


MCF7


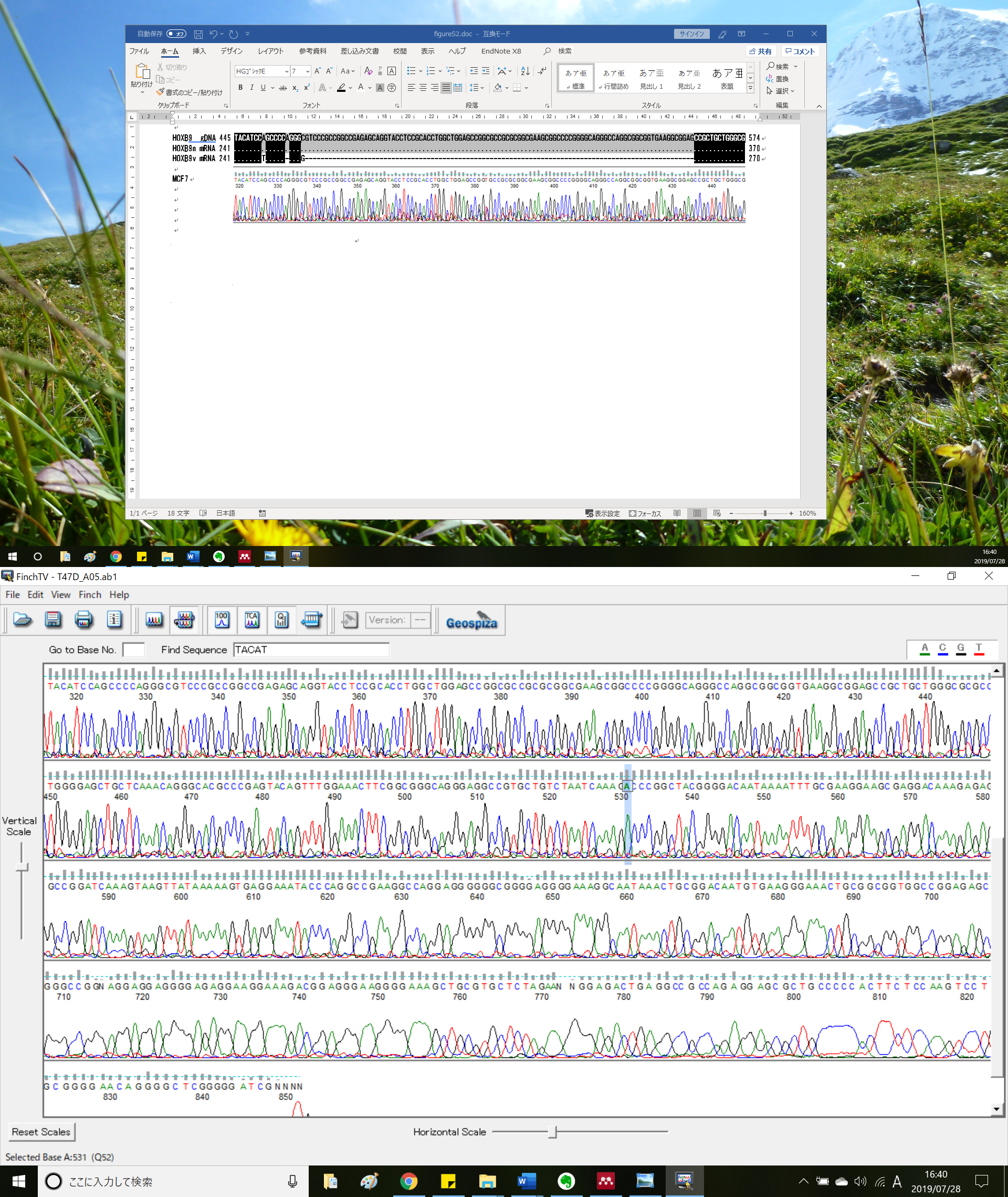


T47D


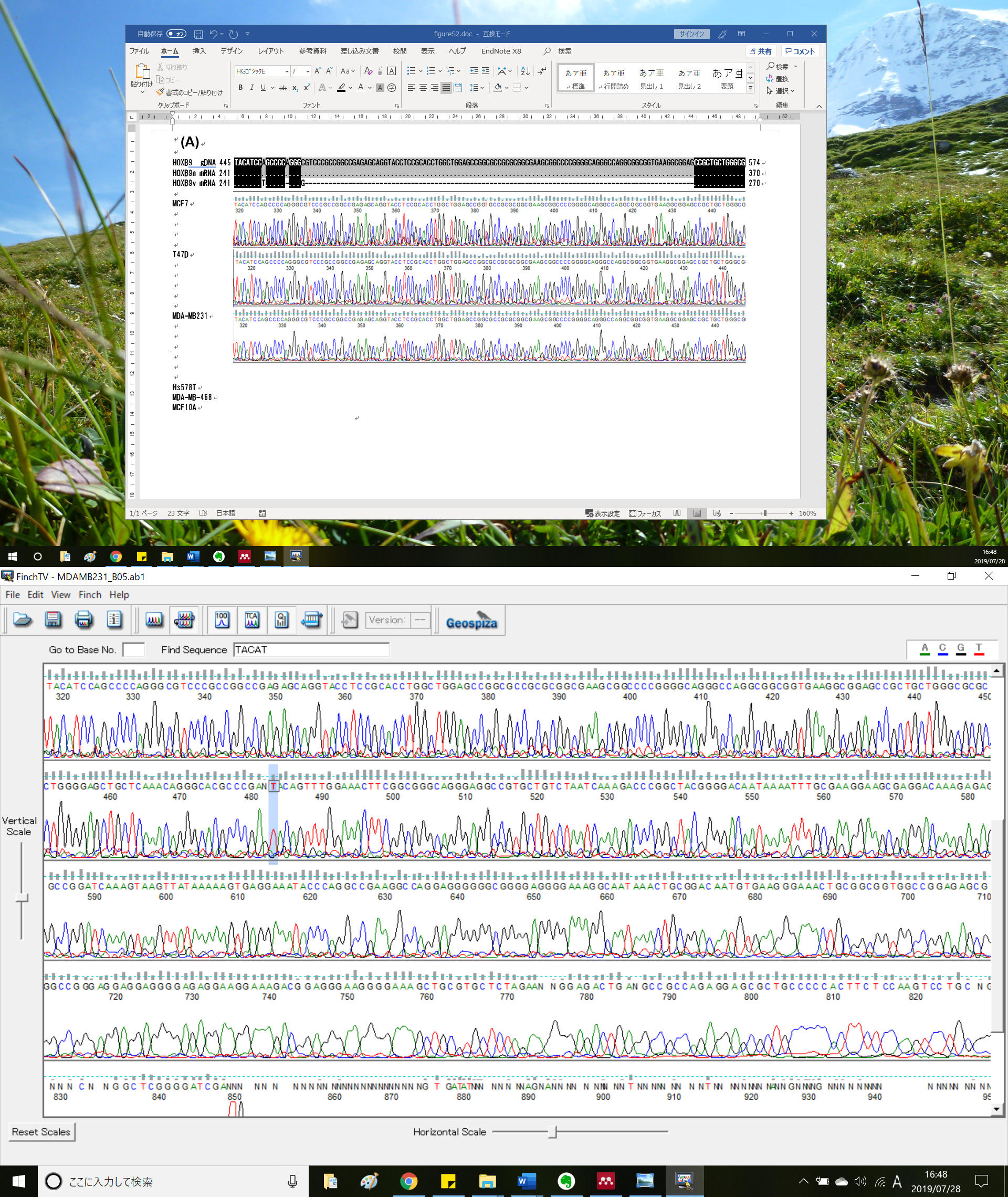


MDA-MB231


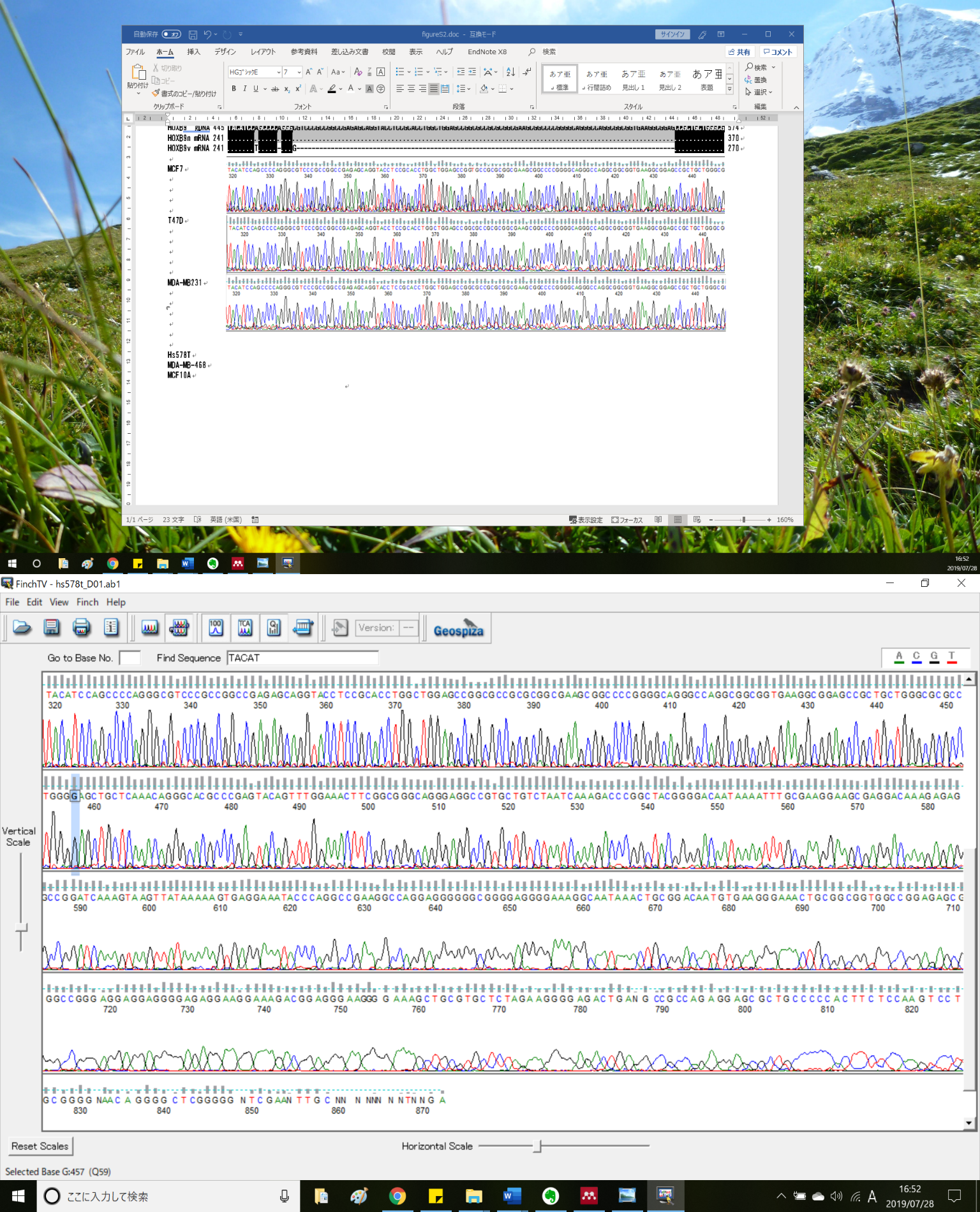


Hs578T


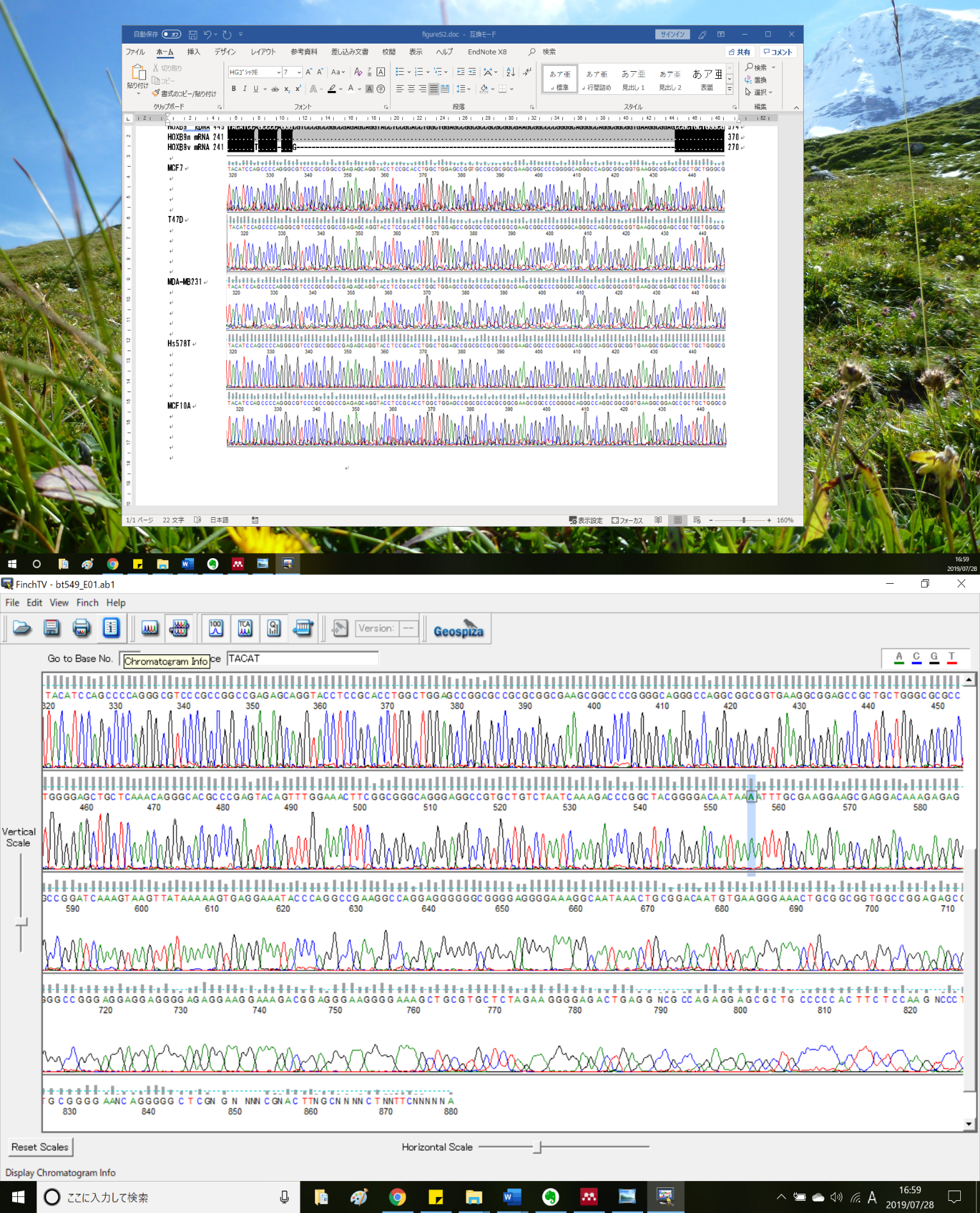


MDA-MB468


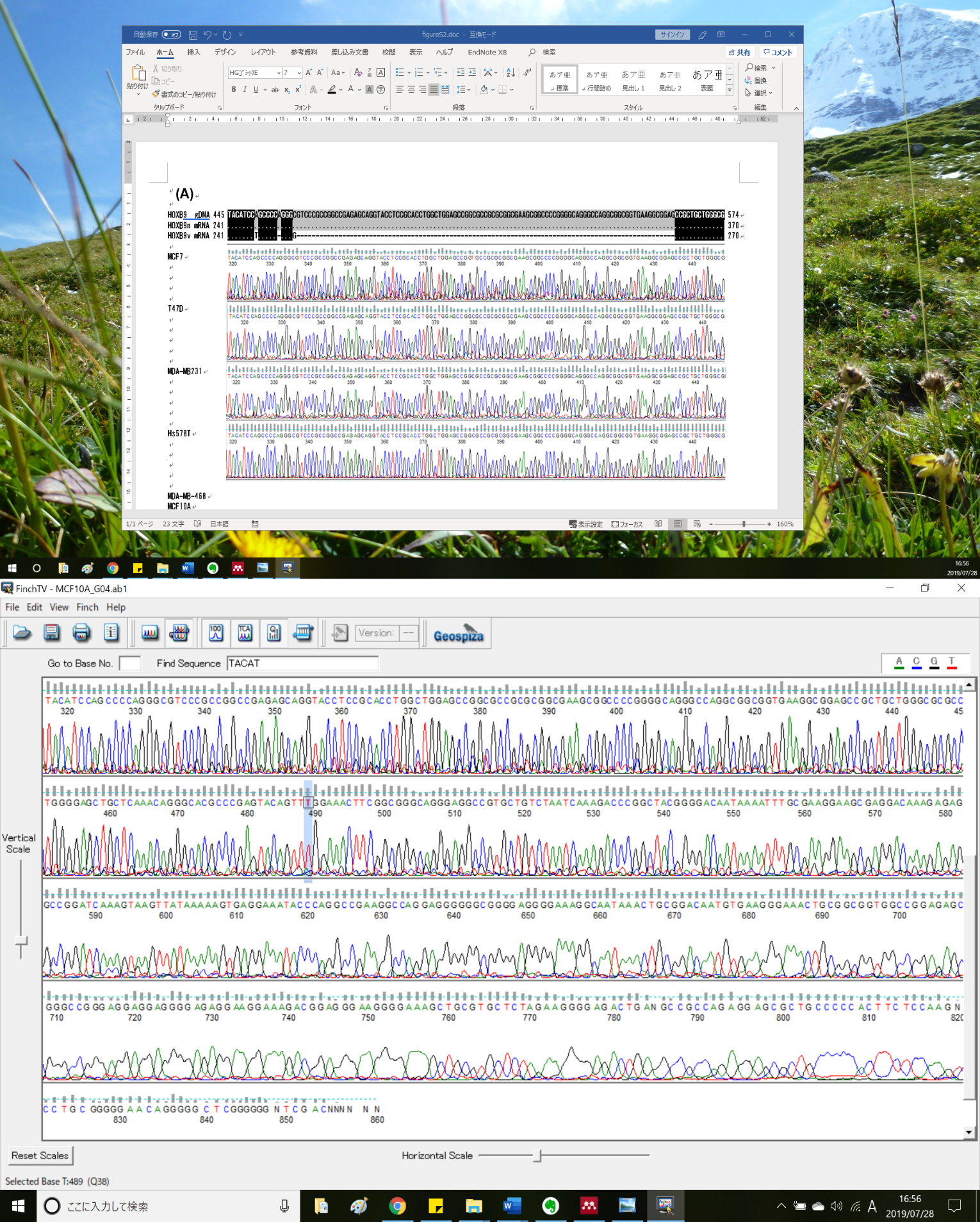


MCF10A
